# Inhibited Production of iNOS by Murine J774 Macrophages Occurs via a *phoP*-Regulated Differential Expression of NF**κ**B and AP-1

**DOI:** 10.1155/2012/483170

**Published:** 2012-07-17

**Authors:** Scott D. Hulme, Paul A. Barrow, Neil Foster

**Affiliations:** School of Veterinary Medicine and Science, The University of Nottingham, Sutton Bonington Campus, Sutton Bonington, Leicestershire, Nottingham NG7 2NR, UK

## Abstract

*Background.* There are no reported data to explain how *Salmonella* suppress nitrite ion production in macrophages or whether this phenomenon is unique to typhoidal or non-typhoidal serovars. The aims of this study were, therefore, to investigate these phenomena. *Methods*. We measured survival of *S. typhimurium* 14028 and its phoP mutant in murine J774 macrophages, cultured with or without interferon gamma. We compared expression of inducible nitric oxide synthase (iNOS) mRNA and protein, and nitrite ion production and also examined binding of nuclear factor *κ*B (NF*κ*B) and activator protein 1 (AP-1) to macrophage DNA. *Results*. *S. typhimurium* 14028 inhibited binding of NF*κ*B and AP-1 to DNA in murine J774. A macrophages via an intact phoP regulon. This correlated with increased survival and reduced iNOS expression. Suppression of NF*κ*B activity was ameliorated in macrophages cultured with IFN-**γ** and this correlated with increased expression of iNOS mRNA and nitrite ion production, although IFN-**γ** had no effect on AP-1/DNA interaction. We show, that with one exception, suppression of iNOS is unique to typhoidal serovars. *Conclusion*. *S. typhimurium* inhibit NF*κ*B and AP-1 interaction with macrophage DNA via the PhoP regulon, this reduces nitrite ion production and is principally associated with typhoidal serovars.

## 1. Introduction

*S. typhimurium* infection in mice is a standard laboratory model for human typhoid, and previous studies have shown that *S. typhimurium* mutants which are unable to survive in murine macrophages are avirulent [1]. Thus, survival of *Salmonella* in macrophages appears to be a critical step in the induction of typhoid. The *Salmonella phoP/phoQ* regulon regulates genes located on *Salmonella* Pathogenicity Island 2 (SPI-2) which encode proteins needed for survival of *Salmonella* inside of macrophages [2] and *Salmonellae* which have mutations in their *phoP/phoQ* regulon are avirulent in mice [3]. The affect of *phoP* on *salmonella* survival is multifaceted but studies by Svensson et al. [4] have shown that *phoP* mutation induces increased nitrite ion production by macrophages compared with nitrite ion production induced by the parent strain but this study did not investigate the mechanisms behind this phenomenon. Studies using iNOS^−/−^ and NADPH^−/−^ mice indicate that reactive nitrogen species (RNS) are important in controlling *Salmonella* later in the infection and this is preceded by a reactive oxygen species (ROS)-dependent control phase [5, 6] and it is also known that nitric oxide increases the sensitivity to cellular acid by *phoP* mutants [7]. Taken together these studies indicate that the ability of *Salmonella* to down-regulate nitrite ion production by host macrophages may be due to the effect of SPI-2 proteins under the control of *phoP* and that this confers survival advantage to the *Salmonella* at some point in the infection, but the underlying inductive mechanism has not been reported. 

For example, nuclear factor kappa B (NF*κ*B) and activator protein-1 (AP-1) are both known to transcribe the iNOS gene [8, 9] but nothing has been reported regarding the activity of these transcription factors in relation to iNOS and in the context of wild type or *phoP* mutant *Salmonella*. Furthermore, there are no reported comparisons between typhoidal and nontyphoidal *Salmonella* serovars with regard to iNOS suppression.

 The aim of this study was to investigate the effect of wild type *Salmonella* and *phoP* mutants on NF*κ*B and AP-1 activity and their subsequent downstream effect using iNOS as a biological readout. We also compared the effect of serovars which cause typhoid in mice with those which do not.

## 2. Materials and Methods

### 2.1. Bacterial Culture and Strains

Bacteria were grown in Luria Bertani (LB) broth (Life Technologies Ltd, Paisley, UK) for 18 h at 37°C under agitation. The bacteria were then subcultured in fresh LB for 4 h to late log phase (established by conventional counts of cfu/mL). Prior to incubation with J774.2 cells, bacteria were adjusted to the multiplicity of infection (moi) of 10. During this study, the following strains/mutants were used: *S. typhimurium* 14028 (ATCC strain), *S. typhimurium* CS022 (*phoP* mutant of 14028, a gift from Dr S. I. Miller, University of Washington, USA), which does not survive in macrophages [10]. In a separate study, the effect of other murine typhoid-inducing (*S. typhimurium* 4/74, *S. enteritidis* KMS1977, *S. dublin* 2229, and *S. choleraesuis* A50) and nontyphoid inducing strains (*S. gallinarum* 9, *S. kedougou* GP, and *S. montevideo* KMS) was analysed. Growth curves for each serovar were obtained as previously stated.

### 2.2. Cell Culture

J774.2 cells were grown to confluence in 96 well plates (Nunc, Naperville, IL, USA) containing RPMI 1640 media at 37°C in CO_2_ (5% v/v). The cells were then washed 3 times in phosphate-buffered saline (PBS), to remove media and nonadherent cells, and incubated in PBS at 22°C for 15 min prior to infection. Cell passages, between 4–16, were used throughout this study. 

### 2.3. Measurement of Nitrite Ion Concentration

Nitrite ion concentration in J774.2 supernatants were measured by Griess reagent kit (Promega, Madison, WI, USA) as per manufacturer instructions. Briefly, 50 *μ*L of supernatant were mixed with 50 *μ*L of sulfanilic solution and incubated at 22°C in darkness for 10 min. 50 *μ*L of napthyl ethylenediamine solution was added and incubated for a further 10 min and the reaction was read on a plate reader (Anthos Labtech Instruments, Hamburg, Germany) at 550 nm. The nitrite ion concentration was determined by comparison with a nitrite ion standard curve with a limit of 1000–5 *μ*M. 

### 2.4. Immunocytochemical Analysis of iNOS Expression

The activity of iNOS in infected and uninfected J774.2 cells was determined by standard immunocytochemical methods. *Salmonella*-infected cells, which had or had not been incubated with 100 U/mL IFN-*γ*, were washed free of media and permeabilised for 10 min at ambient temperature in 0.05% v/v Triton X-100 after 2, 7, 12, and 24 h postinfection. The cells were then fixed for 15 min in 5% v/v paraformaldehyde at ambient temperature and washed in PBS. Fixed cells were then incubated in the dark on an end-to-end shaker for 60 min at 4°C with mouse anti-iNOS IgG (Autogen Bioclear, UK) PBS-Tween 20 (0.02% v/v Tween, PBS-T) to give a final antibody concentration of 1/100. The cells were then washed three times in PBS-T and incubated in identical conditions but with secondary anti-mouse antibody conjugated to fluorescein isothiocyanate (FITC, Sigma). Samples were viewed on a TCS NT confocal laser scanning microscope equipped with an argon laser (Leica, Cambridgeshire, UK). Controls included J774.2 cells which had not been infected or incubated with IFN-*γ* and also uninfected cells which were incubated with 100 U/mL IFN-*γ*. 

### 2.5. Western Blotting

Expression of iNOS protein in *Salmonella*-infected J774.2 cells was assessed by western blotting. J774 cells were lysed in cold water containing protease inhibitors (pepstatin 1 *μ*g/mL; phenylmethylsulphonyl fluoride 1 mM and leupeptin 10 *μ*M) (Sigma) and protein concentration was determined using a bicinchoninic acid (BCA) assay (Pierce, Cheshire, UK). 10 *μ*g/mL J774.2 protein was electrophoresed by standard methods on two 10% acrylamide gels ran back-to-back. To test for parity in protein loading, one gel was stained with Coomassie blue whilst proteins on the second gel were transferred to Hybond-C nitrocellulose paper (Amersham, Buckinghamshire, UK) by a Trans-blot SD, semi-dry transfer cell (Bio-Rad, Hertfordshire, UK). The nitrocellulose was blocked at room temperature for 60 min in PBS-T containing bovine serum albumin (5% w/v, Sigma). After blocking, the nitrocellulose was washed 3 times for five min in PBS-T on an end-to-end shaker at room temperature. The nitrocellulose was then incubated for 60 min in PBS-T containing 1/200 dilution of anti-mouse iNOS (Autogenbioclear, UK), washed three times as before and incubated for 60 min at room temperature in PBS-T containing Rabbit anti-mouse IgG conjugated to horseradish peroxidase (Sigma, 1/12000 dilution). The nitrocellulose was then washed and developed using an enhanced 3′, 3′ diaminobenzidine (DAB) kit (Vector Laboratories, Burlingame, CA, USA). 

### 2.6. iNOS mRNA Expression

iNOS expression in J774.2 cells infected with *Salmonella* was measured by reverse transcription polymerase chain reaction (RT-PCR) using a previously reported method [11]. Briefly, 6 × 10^6^ J774 cells were suspended in 3 mL TRI reagent (Sigma) and stored at –70°C until required (used within 14 days). Samples were centrifuged at 12,000 g for 10 min in a bench top centrifuge at 4°C. The supernatants were transferred to separate tubes, and 200 mL chloroform was added per mL TRI reagent prior to incubation for 10 min at 22°C. The sample was then centrifuged at 12,000 g and 4°C for 15 min, the aqueous phase was removed, and an equal volume of propan-2-ol was added. The sample was centrifuged at 12,000 g for 10 min, and the RNA pellet was washed in a mixture of 1 vol 75% ethanol : 1 vol sterile water. The mixture was then centrifuged for 10 min at 7,500 g, and, after removal of the supernatant, the pellet was allowed to air dry for further 10 min. The pellet was then resuspended in diethyl pyrocarbonate treated water. RNA purity was measured using an Ultraspec III spectrophotometer (Pharmacia, Hertfordshire, UK) and was found to have a typical 260/280 nm ratio of 1.9 to give yields of around 100 *μ*g/mL. RNA concentration was adjusted to 1 *μ*g/*μ*L prior to the RT reaction. RT reactions were performed on a thermal cycler (Eppendorf, Hamburg, Germany) using the following previously reported [11] primer sequences:

 Forward: 5′-GTA AAC TGC AAG AGA ACG GAG AAC-3′.
 Reverse: 3′-GAG CTC CTC CAG AGG GGT AG-5′.

As a loading control, the following glyceraldehyde-3-phosphate dehydrogenase (GAPdh) primer sequences were used:

 Forward: 5′-GTT CAG CTC TTG GAT GAC CTT GCC-3′.
 Reverse: 3′-TCC TGC ACC ACC AAC TGC TTA GCC-5′.

#### 2.6.1. Preparation of Nuclear Lysates

 Nuclear lysates were prepared by scraping cells into 5 mL PBS and washing once with buffer I (HEPES 10 mM, KCl 10 mM, MgCl_2_ 1.5 mM, pH 7.9). The cells were disrupted by freeze-thawing twice in 1 mL buffer I containing Nonidet P-40 (NP-40) (5%, Sigma). All subsequent procedures were carried out at 4°C. The lysate was centrifuged for 5 min at 2000 rpm in a bench-top microfuge. The pellet was then washed twice with buffer I and NP-40 before being centrifuged at 12,000 rpm for 5 min to obtain the nuclear pellet. Nuclear proteins were extracted from the pellet for 10 min with 30–40 *μ*L extraction buffer (HEPES 20 mM, NaCl 420 mM, MgCl_2_ 1.5 mM, EDTA 0.2 mM, 25% glycerol, pH 7.9). After mixing, the suspension was centrifuged twice as stated above. The supernatant was then diluted in 40–60 *μ*L dilution buffer (HEPES 20 mM, KCl 50 mM, EDTA 0.2 mM, glycerol 20%, pH 7.9). Protease inhibitors (pepstatin 1 *μ*g/mL, phenylmethylsulphonyl fluoride 1 mM, leupeptin 10 *μ*M, Sigma) were added to the lysate which was then used immediately or stored at –70°C for up to 14 days.

#### 2.6.2. Electromobility Shift Assay (EMSA)

 EMSA was used to determine DNA binding of NF*κ*B (p50) and activating protein (AP)-1 (c-Jun) to J774.2 DNA following infection by *Salmonella* and/or incubation with IFN-*γ* (100 U/mL). EMSA reactions were performed using a kit as per manufacturer instructions (Promega, USA) using the following oligonucleotide sequences:

AP-1 (c-Jun)

 5′-CGC TTG ATG AGT CAG AAG GAA-3′
 3′-GCG AAC TAC TCA GTC GGC CTT-5′

NF*κ*B (p50)

 5′-AGT TGA GGG GAC TTT CCC AGG C-3′
 3′-TCA ACT CCC CTG AAA GGG TCC G-5′

Kit controls included HeLa cell nuclear lysate with consensus oligo (positive control) and HeLa cell nuclear lysate without consensus oligo (negative control). As an additional negative control, J774.2 cells which had not been infected or incubated with IFN-*γ* were used.

Digital Image Analysis was performed using a Phoenix 1D analyser using a power scanner V.3 (Phoretix, Newcastle upon Tyne, UK).

### 2.7. Statistical Analysis

Mann-Whitney analysis (Minitab) was used to measure significant difference at the 95% confidence limit between different test groups and between the same test groups at different time points.

## 3. Results

### 3.1. The Effect of *Salmonella phoP* on Survival and Nitrite Ion Production in Murine J774.2 Cells

**Figure 1:**
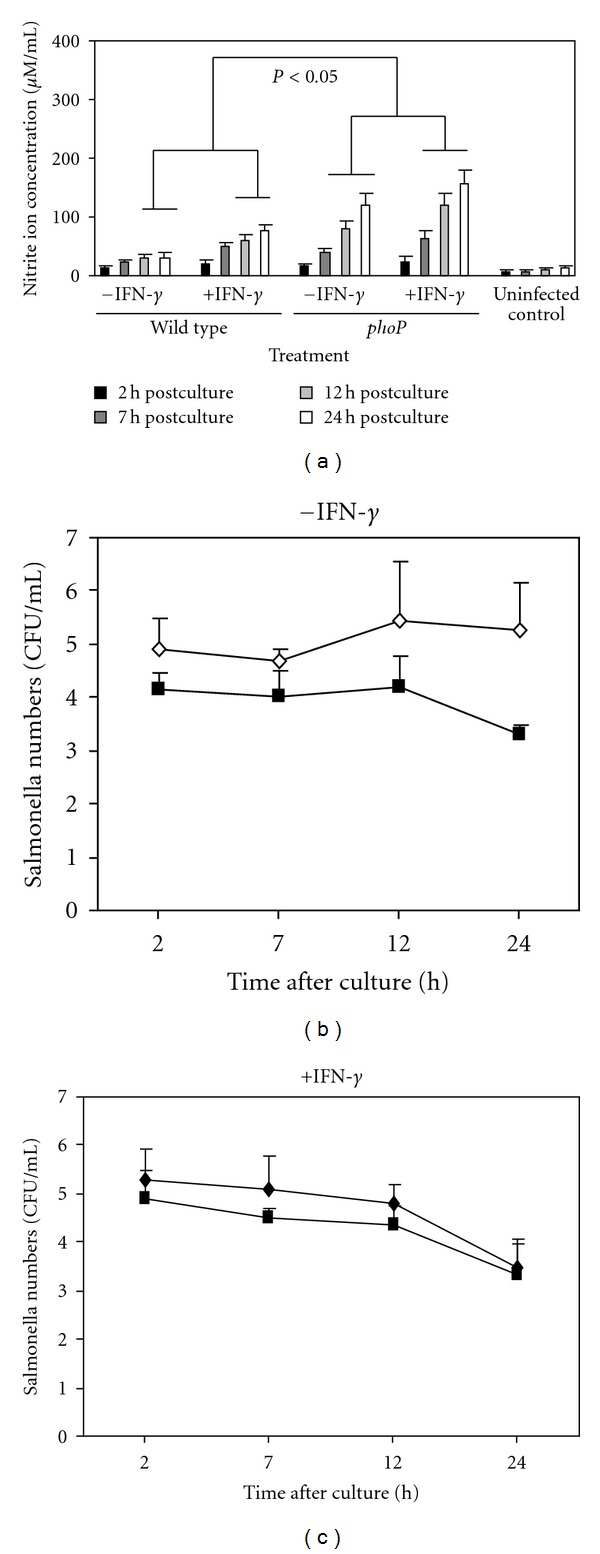
Wild type *S. typhimurium* 14028 suppresses macrophage nitrite ion production via the *phoP *regulon but is inhibited by IFN-*γ*. Histograms show nitrite ion concentrations measured in supernatants from macrophages cultured with 14028 wild type and *phoP* mutants and uninfected controls and with or without the addition of IFN-*γ*. *Significant increase (*P* < 0.05) in nitrite ion concentration in cell supernatants compared to uninfected controls at equivalent time points. Significant increase (*P* < 0.05) in nitrite ion concentrations recovered from macrophage supernatants cultured with 14028 *phoP* mutants compared to 14028 wild type is also shown. Each point (and standard deviations) is mean values calculated from triplicate cultures replicated on 5 separate occasions.

Nitrite ion production by macrophages was significantly (*P* < 0.05) lower when the cells were cultured with wild type *S. typhimurium* 14028 compared to nitrite ion production by macrophages cultured with 14028 *phoP* mutant. However addition of IFN-*γ* to culture media significantly increased (*P* < 0.05) nitrite ion concentrations in supernatants recovered from both wild type and *phoP*-cultured macrophages (Figure 1(a)).

The numbers of wild type 14028 recovered from macrophages were also between 1-2 logs greater after 12–24 h culture compared with the numbers of 14028 *phoP* mutants recovered from cells over the same period (Figure 1(b)). When macrophages were cocultured with IFN-*γ*, the numbers of wild type 14028 recovered from cells decreased and were comparable to the numbers of 14028 *phoP* mutants recovered over the same time period (Figure 1(c)).

### 3.2. The Effect of *Salmonella phoP* on iNOS mRNA and iNOS Protein Expression by J774.2 Cells

**Figure 2:**
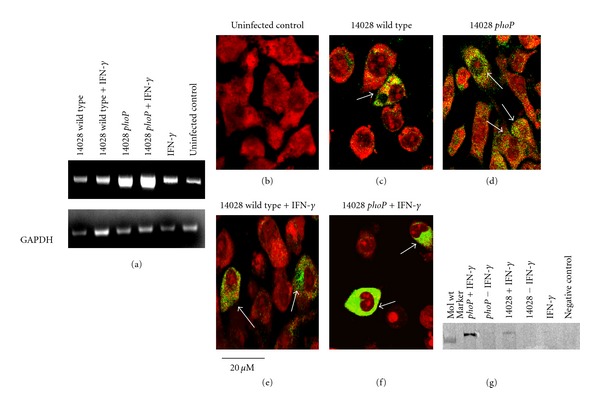
*S. typhimurium* 14028 *phoP* regulon suppresses iNOS mRNA and protein expression in murine macrophages but fails to suppress iNOS when co-cultured with IFN-*γ*. (a) PCR showing iNOS mRNA expression is reduced in macrophages cultured with 14028 wild type compared to 14028 *phoP* mutants. The addition of IFN-*γ* to culture media increased iNOS mRNA expression in macrophages infected with either wild type or *phoP* mutant. (b) Intracellular iNOS protein cannot be detected by immunocytochemistry in uninfected (control) macrophages. (c) Suppression of intra-cellular iNOS protein observed in macrophages cultured with wild type 14028 compared to macrophages cultured with *phoP* mutants (d). Addition of IFN-*γ* to cell culture media also increased intra-cellular iNOS in macrophages cultured with wild type 14028 (e) or *phoP* mutants (f). Arrows show iNOS positive cells, scale bar bottom left: 20 *μ*m. (g) Western blot of iNos protein in whole cell preparations stimulated with wild type 14028 or 14028 *phoP* with or without IFN-*γ*. Lane 1, molecular weight marker: 130 kDa. Lane 7: Unstimulated J774 cells (negative control). All analyses are representative of data obtained on 3 separate occasions.

Increased iNOS mRNA expression was detected in macrophages cultured with 14028 *phoP* mutants when compared with 14028 wild type *Salmonella* and in both cases the iNOS mRNA signal was increased by coculture with IFN-*γ* (100 U/mL) (Figure 2(a)). However, when adjusted for GADPH expression, iNOS mRNA expression, in J774 cells, induced by IFN-*γ* and 14028 wild type, was still slightly lower than was induced by IFN-*γ* alone.

Confocal microscopy data also clearly showed that a *phoP* mutation (Figure 2(d)) caused a more intense iNOS protein signal in the cytoplasm of J774.2 cells when compared to uninfected control macrophages (Figure 2(b)) or wild type 14028-infected cells (Figure 2(c)). The intensity of iNOS signal was increased by co-culture of macrophages with wild type 14028 and IFN-*γ* (Figure 2(e)) and was even more intense in macrophages which were co-cultured with 14028 *phoP* mutants and IFN-*γ* (Figure 2(f)). The results obtained by confocal were also repeated by Western blotting (Figure 2(g)) and in this case J774 cells co-cultured with 14028 wild type and IFN-*γ* produced a greater amount of iNOS protein when compared to J774 cells cultured only with IFN-*γ*.

### 3.3. The Effect of *phoP* Mutation on NF*κ*B and AP-1 Binding to Macrophage DNA

**Figure 3:**
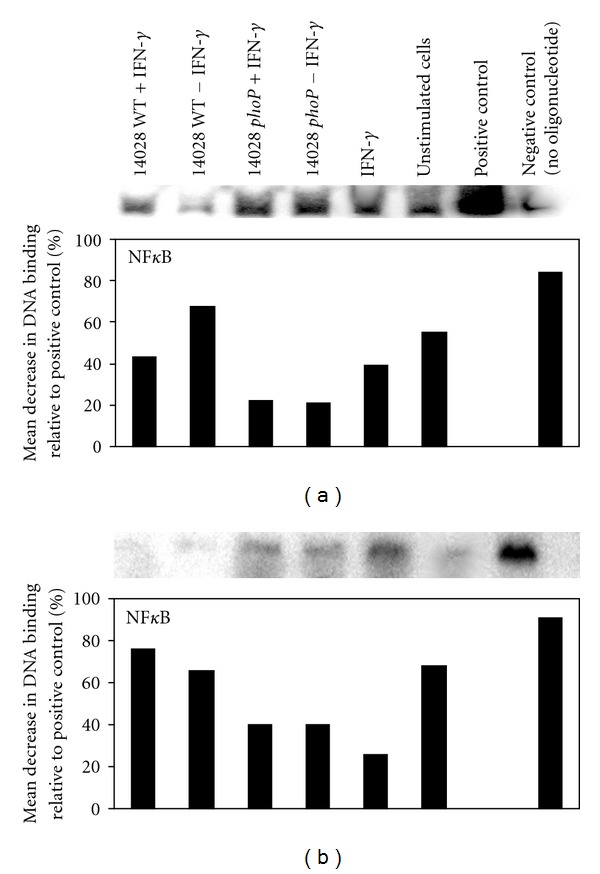
Wild type *S. typhimurium* suppress NF*κ*B/DNA interaction in J774.2 cells 2 and 12 h afterculture via *phoP*. (a) 2 h postculture. (b) 12 h post-culture. Controls include, uninfected J774.2 cells, manufacturers kit positive control (HeLa cell nuclear lysate with oligonucleotide), and manufacturers kit negative control (HeLa cell nuclear lysate without oligonucleotide). Data is representative of EMSAa performed on 3 separate occasions.

Our results show that mutation in the *Salmonella phoP* regulon increased the amount of NF*κ*B and AP-1 bound to macrophage DNA after 2 h, when compared to wild type 14028 (Figure 3(a)). When compared to a positive control, NF*κ*B/DNA interaction following wild type 14028 infection was reduced by a mean of 68% but when infected macrophages were co-cultured with IFN-*γ* (100 U/mL), NF*κ*B/DNA interaction was reduced by 42% (Figure 3(a)). In comparison, when macrophages were infected with 14028 *phoP* mutants, NF*κ*B/DNA interaction was only reduced by a mean of 22% (relative to the positive control) and this remained constant following co-culture of infected cells with IFN-*γ* (Figure 3(a)). After macrophages were cultured with wild type 14028 for 12 h, NF*κ*B/DNA interaction was reduced by a mean of 66% but when infected macrophages were co-cultured with IFN-*γ*, NF*κ*B/DNA interaction was reduced by 76% (Figure 3(b)). when macrophages were infected for 12 h with 14028 *phoP* mutants, NF*κ*B/DNA interaction was only reduced by 40% (relative to the positive control) and this also remained constant following co-culture of infected cells with IFN-*γ* (Figure 3(b)).

**Figure 4:**
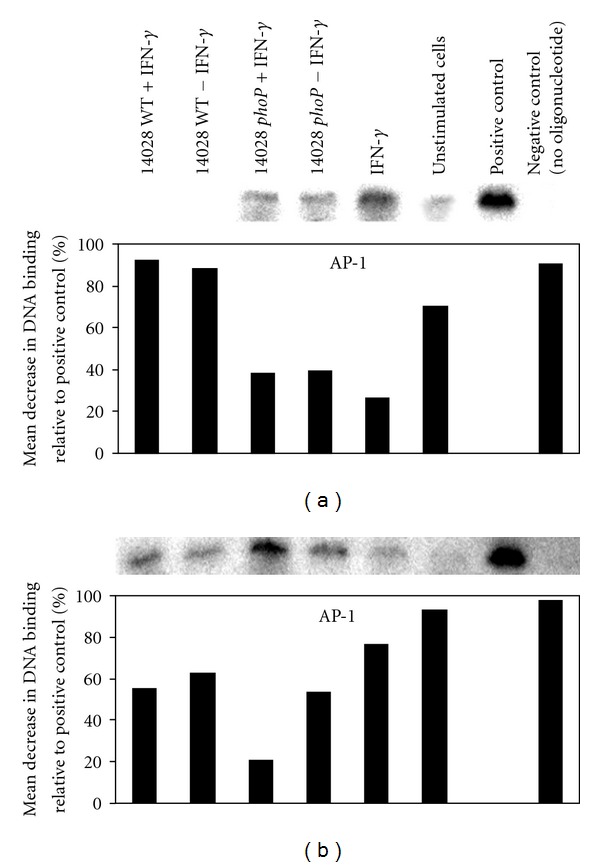
Wild type *S. typhimurium* suppresses AP-1/DNA interaction in J774.2 cells 2 and 12 h afterculture via *phoP*. (a) 2 h postculture. (b) 12 h postculture. Controls include, uninfected J774.2 cells, manufacturers kit positive control (HeLa cell nuclear lysate with oligonucleotide), and manufacturers kit negative control (HeLa cell nuclear lysate without oligonucleotide). Data is representative of EMSAa performed on 3 separate occasions.

In macrophages infected for 2 h with wild type 14028, results obtained for AP-1/DNA binding was equivalent to those obtained for a negative control which contained no oligonucleotide (92% reduction in DNA binding compared to the positive control) and this was altered very little by coculture with IFN-*γ* (88% reduction compared to the positive control) (Figure 4(a)). However, when macrophages were infected with 14028 *phoP* mutants for 2 h, AP-1/DNA binding was only reduced by 39% relative to the positive control and this level was maintained following co-culture with IFN-*γ* (Figure 4(a)).

After macrophages were cultured with wild type 14028 for 12 h, AP-1/DNA interaction was increased with a reduction relative to the positive control of 62.9% and AP-1/DNA interaction was increased further following co-culture of infected macrophages with IFN-*γ* for 12 h, in which a mean reduction of 55% (relative to the positive control) was measured (Figure 4(b)). However, AP-1/DNA interaction in macrophages infected with 14028 *phoP* mutants for 12 h was reduced only by 53% compared to the positive control but, following co-culture with IFN-*γ*, AP-1/DNA interaction was increased with a mean reduction of only 21% relative to the positive control (Figure 4(b)).

### 3.4. *Salmonella* Serovars Which Induce Murine Typhoid Suppress Nitrite Ion Production

**Figure 5:**
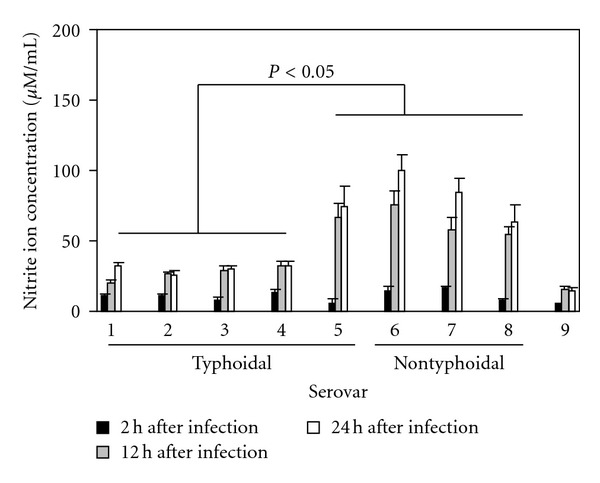
Nitrite ion production by J774 cells cultured with different typhoid-causing or non-typhoid-causing *Salmonella* serovars and co-cultured with IFN-*γ*. 1: *S. typhimurium* 14028; 2: *S. typhimurium* 4/74; 3: *S. enteritidis* KMS 1977; 4: *S. dublin* 2229; 5: *S. choleraesuis* A50; 6: *S. kedougou* GP; 7: *S. montevideo* KMS; 8: *S. gallinarum*; 9: uninfected cells. All experiments were replicated 3 times on at least 5 separate occasions. Statistical bars show significant different (*P* < 0.05) between nitrite ion production in macrophages infected with typhoidal and nontyphoidal serovars.

When macrophages were cultured with *Salmonella* serovars which are known to induce murine typhoid, nitrite ion concentrations were reduced in cell supernatants compared to supernatants isolated from macrophages cultured with nontyphoidal strains over the same time period (Figure 5). However *S. Choleraesuis*, which is known to induce murine typhoid, was an exception to this rule since it stimulated nitrite ion production by macrophages at similar levels to those measured in supernatants isolated from macrophages cultured with nontyphoidal serovars (Figure 5).

## 4. Discussion

Eriksson et al. [12] have previously reported that hyper-survival mutants of *S. typhimurium* TT16729, obtained by multiple passage through J774 cells, induced lower nitrite ion concentrations than did the parent strains, although an attenuated *Salmonella* mutant was not studied for comparison. However, Svensson et al. [4] reported that wild type *S. typhimurium* 14028 induced lower nitrite ion production in murine bone marrow derived macrophages when compared with an *S. typhimurium* constitutive *phoP* ^c^ mutant but that the survival of the *phoP* was only marginally different (<0.5 Log) to the 14028 wild type. In contrast to this result, we show that there is a clear increase in survival rates of wild type 14028 compared to its *phoP* mutant in J774.2 macrophages. However, the differences we observe may conceivably be due to differences in cell type or moi, since our moi was constant at 10 : 1 whereas the previous study used 14028 at an moi of 15 : 1 and *phoP* ^c^ at an moi of 17 : 1 [4]. In contrast to these studies, a study by Das et al. [13] has shown that in the murine macrophage cell line RAW264.7, wild type *Salmonella* inhibit IFN-*γ*-induced NO production via the virulence gene nirC and that this correlates with increased cellular survival. However our study shows that iNOS mRNA, iNOS protein, and nitrite ion production are increased when wild type 14028-infected J774 cells are co-cultured with IFN-*γ* but overall wild type infected cells elicit much weaker responses than do 14028 *phoP*-infected macrophages, and this occurs in the presence or absence of IFN-*γ*. We have also found that 14028 wild type and 14028 *phoP* have comparable sensitivity to exogenous nitrite ions, as shown by bacterial growth curves obtained at different nitrite ion concentration (data not shown). It is possible that the inhibition of NO production via iNOS suppression may have relevance later in the infection as shown by Mastroeni et al. [5] or it is also possible that the inhibition of iNOS per se (rather than downstream NO) may have immediate impact on other factors. For example, iNOS-dependant induction of 8-nitroguanosine 3′,5′-cyclic monophosphate (8-nitro-cGMP) has been shown to induce heme oxygenase 1 (HO-1) which has both cytoprotective and antimicrobial effects in murine Salmonellosis [14]. NO production may also enhance other antimicrobial pathways, for example, via interaction with reactive oxygen species [15]. 

Our study also attempted to relate changes in iNOS to the activity of two critical transcription factors (NF*κ*B and AP-1) in infected J774 macrophages, with or without co-culture with IFN-*γ*. The iNOS gene has previously been shown to be transcribed by nuclear factor kappa B (NF*κ*B) (p50/65) [16] which may translocate to the cell nucleus following stimulation by interferon gamma (IFN-*γ*) [17, 18] and this may act synergistically with bacterial lipopolysaccharide (LPS), depending on relative concentrations [19]. 

As well as NF*κ*B binding sites, promoter sequences on murine iNOS genes also contain binding sites for AP-1 and IFN-*γ* response elements (*γ*-IRE) ([8] reviewed [20]). However, AP-1 activation during *Salmonella* invasion of macrophages has not been comprehensively studied but temporal changes in the heterodimeric composition of AP-1 during culture of murine macrophages with *S. typhimurium* LPS or porins have been reported [21]. 

 Our data was interesting for a number of reasons; firstly we show that wild type 14028 suppresses NF*κ*B/DNA interaction within the first 2 h postinfection of J774 macrophages but this is not the case when *phop* mutation is induced. This suggests that the ability to suppress NF*κ*B activity is, therefore, *phoP*-dependent. However, the inherent suppression of NF*κ*B activity by wild type 14028 is overcome when the macrophages are co-cultured with IFN-*γ*, and this is consistent with one study which has shown that IFN-*γ* suppresses *phoP* transcription in wild type *Salmonella* [22]. Interaction of NF*κ*B with macrophage DNA was also reduced further after 12 h culture with wild type 14028 and co-culture with IFN-*γ* had no effect, whereas *phoP* mutants maintained relatively strong NF*κ*B/DNA interaction (although this was also reduced by about half when compared to NF*κ*B/DNA interactions measured after 2 h). These results suggest that an intact *phoP* regulon promotes significant changes in the ability of NF*κ*B to interact with DNA and this probably had a significant impact on the iNOS suppression we observed in wild type-infected cells. Saura et al. [23] have shown that IFN-*γ* induces nuclear translocation of interferon regulatory factor 1 (IRF-1) which then synergises with NF*κ*B to transcribe iNOS. Therefore, it is possible that this is an additional mechanism by which IFN-*γ* increases iNOS transcription in our study, as well as the increased NF*κ*B/DNA interaction we have shown.

However, our study also indicates that there is temporal separation between the induction of NF*κ*B/DNA interaction and AP-1/DNA interaction in 14028 wild type infected macrophages but this was not observed in macrophages infected with 14028 *phoP* mutants. After 2 h postinfection with wild type 14028, no discernable interaction between AP-1 and macrophage DNA was measured but after 12 h post-infection AP-1/DNA interaction was measured and this was increased when the cells were co-cultured with IFN-*γ*, although this was on average 55–63% lower than that measured for the positive control. In contrast, when the macrophages were cultured with 14028 *phoP* mutants, AP-1/DNA interaction was only about 53% lower than the positive control but when the cells were co-cultured with IFN-*γ*, AP-1/DNA binding was only 21% lower than the positive control. These results also indicate that an intact *phoP* regulon prevents long-term interaction of AP-1 with DNA and IFN-*γ*-induced increase in AP-1/DNA interaction (as was the case with NF*κ*B). We, therefore propose that the affect of the *phoP* regulon to prevent long-term and strong interaction of macrophage DNA with NF*κ*B and AP-1 and IFN-*γ*-stimulated upregulation of this interaction will have a profound effect on iNOS expression and nitrite ion production via reduced exposure of iNOS promoter sequences within macrophage DNA to these essential transcription factors.

No previously published data exists which has compared iNOS activity and nitrite ion production in murine macrophages cultured with typhoidal and non-typhoid *Salmonella* serovars. However, Eisenstein et al. [24] have shown that *S. typhimurium* and *S. dublin* both inhibit nitrite ion production by murine splenocytes, and although this study at least considered different typhoid inducing serovars, a comparison between typhoid-inducing and non-inducing serovars was not reported. The one surprising exception in our study was *S. choleraesuis* which failed to down- regulate nitrite ion production by macrophages. *S. choleraesuis* causes typhoid-like systemic disease in a much wider range of mammalian hosts than do *S. typhimurium*, *S. Dublin,* or *S. enteritidis* [25] and we cannot, as yet, explain why this serovar does not down regulate iNOS. However, all other typhoid-inducing serovars were able to down-regulate nitrite ion production by murine macrophages whereas those which do not induce typhoid were unable to do so. This may suggest that the ability of typhoid-inducing *Salmonella* may allow dissemination to deeper tissues, via a *phoP*-induced suppression of NF*κ*B and AP-1 and subsequent suppression of iNOS.

Our data may be relevant in the future treatment of Typhoid in humans, since it suggests a possible role for the adjunctive use of IFN-*γ* (and antibiotic) to overcome *phoP-*dependent iNOS suppression. Thus, increasing nuclear translocation of essential transcription factors needed for transcription of the iNOS gene and nitrite ion production.
